# An extramedullary plasmacytoma in the kidney of a 14-year-old girl: Case report and review of the literature

**DOI:** 10.1097/MD.0000000000006092

**Published:** 2017-02-10

**Authors:** Yan-hui Mei, Jian-peng Yu, Gang Li

**Affiliations:** a Department of Urology, Affiliated Hospital of Binzhou Medical University, Binzhou; b Department of Urology, Second Hospital of Tianjin Medical University, Tianjin Institute of Urology, Tianjin, China.

**Keywords:** extramedullary plasmacytoma, malignant tumor, renal tumors

## Abstract

**Rationale:**

Extramedullary plasmacytoma (EMP) a rare plasma cell disorder and is frequently associated with plasma cell bone marrow infiltration. Most EMPs involve mucosal lymphoid tissue, especially in the nasopharyngeal area, respiratory tract, and head and neck region. Primary involvement of the kidney is exceedingly rare.

**Patient Concerns:**

A 14-year-old girl was admitted in our hospital with intermittent right upper quadrant pain for 1 month and recent (1 day) progressive deterioration. There was a mass found by ultrasonography in the right kidney and subsequent abdominal computed tomography scan revealed a 3 cm mass within the right kidney.

**Diagnoses:**

Pathology revealed typical histology of plasmacytoma and immunohistochemistry revealed the expression of CD138, CD45, vimentin, and Kappa light chain.

**Interventions:**

The patient successfully underwent radical nephrectomy with an uneventful recovery. She received no chemotherapy or radiotherapy after surgery.

**Outcomes:**

There was no recurrence or metastasis during a 22-month follow-up.

**Lessons:**

Our case study demonstrated that renal EMP with a relatively indolent clinical course, if detected at an early stage, can be treated by radical nephrectomy without adjuvant therapy. Generally, the clinical outcome and prognosis of EMP are favorable

## 1 Introduction

Solitary extramedullary plasmacytoma (EMP) is a rare plasma cell disorder without bone marrow involvement. The occurrence of plasmacytoma has a male-to-female ratio of 3:1 and constitutes about 3% of all plasma cell tumors.^[1]^ Most EMPs involve mucosal lymphoid tissue, especially in the nasopharyngeal area, respiratory tract, and head and neck region.^[2]^ About 15% of plasmacytomas may progress to multiple myeloma (MM).^[3]^ No clear therapeutic guidelines are available due to its rarity. In this case report and short review, the patient's characteristics, diagnosis, treatment, and outcome are discussed.

## 2 Case report

**Figure 1:**
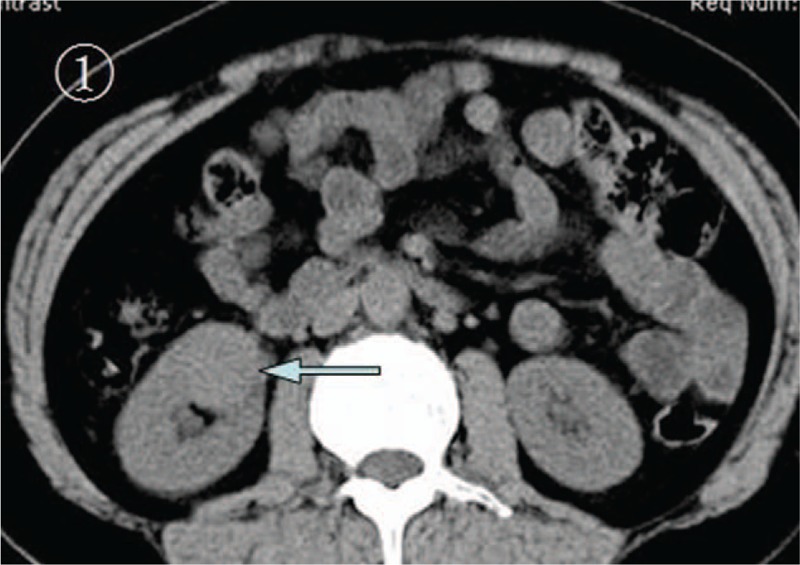
The abdominal precontrast CT scan. This scan revealed a mass that was measured about 3 cm at the hilum of the right kidney (arrow). CT = computed tomography.

**Figure 2:**
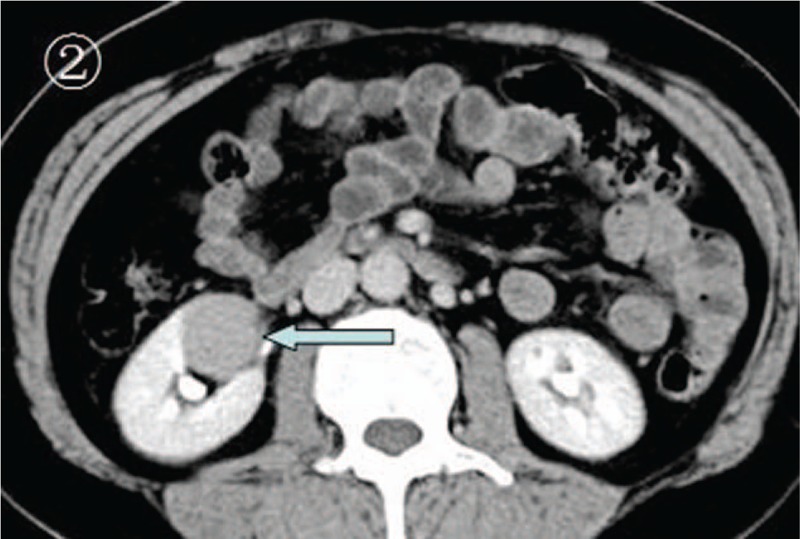
The abdominal postcontrast enhanced CT scan. This scan revealed no obvious enhancement of the mass, but showed a clear demarcation of the tumor from its surrounding normal tissues and a regular shape of the tumor (arrow). CT = computed tomography.

**Figure 3:**
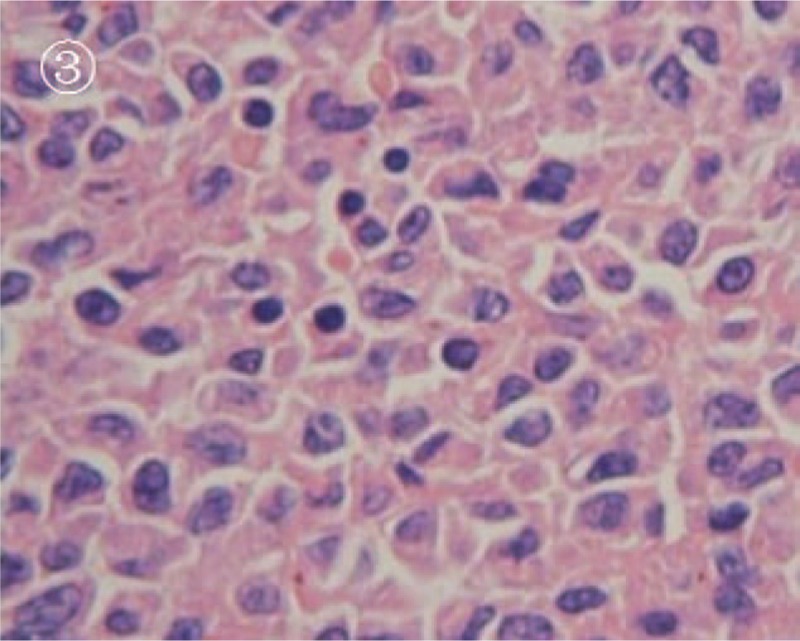
The photomicrograph of HE staining. It was shown that the tumor was composed of round monomorphic cells with vesicular and eccentric nuclei and immature plasma cells (magnification, ×400). HE = hematoxylin and eosin.

**Figure 4:**
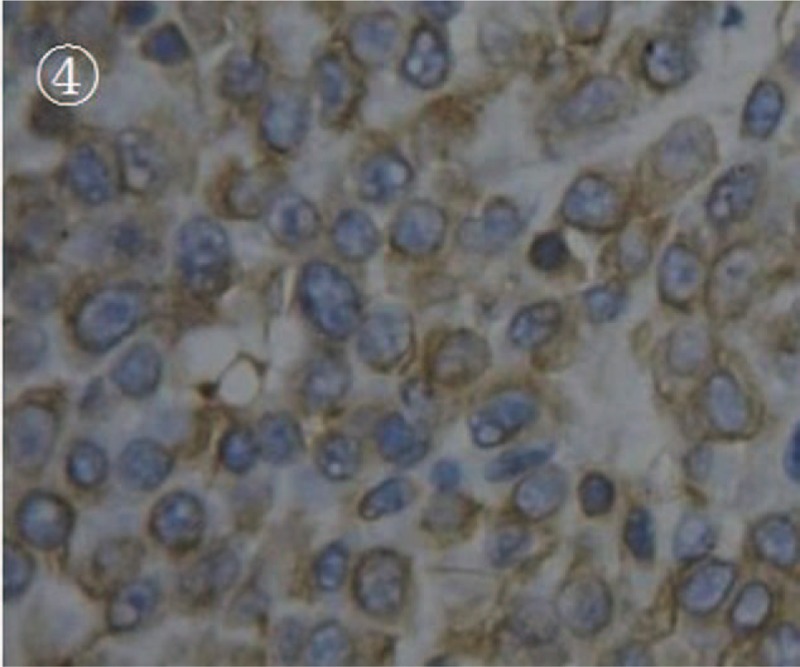
The photomicrograph of vimentin immunohistochemical staining. It was shown that the tumor cells were positively stained with vimentin antibody (magnification, ×400).

A 14-year-old girl was referred to our hospital for intermittent right upper quadrant pain for 1 month and recent (1 day) progressive deterioration. There were no unusual findings on routine physical examination. Before the diagnosis, she had no gross hematuria. Results from routine medical laboratory tests including clinical biochemistry showed normal values except for macroscopic hematuria. A conventional radiographic bone and chest examination also yielded no abnormal findings. Computed tomography (CT) scan of the abdomen revealed a homogeneous and well-circumscribed mass, measuring about 3 cm, adjacent to the hilum of the right kidney (Fig. 1). CT attenuation of this tumor was approximately 50 HU. Meanwhile, renal parenchymal structure was normal and no enlarged lymph nodes were detected. Contrast-enhanced CT scan showed no significant enhancement of the tumor mass (Fig. 2). Therefore, on the basis of these findings, a preliminary clinical diagnosis of renal cell carcinoma was made. The patient then successfully underwent radical nephrectomy with an uneventful recovery. Microscopic examination of tumor section showed that the tumor was composed of round monomorphic cells with vesicular and eccentric nucleus and immature plasma cells (Fig. 3). The tumor cells had irregularly shaped nuclei that varied in size and shape. The nuclei were surrounded by variable amounts of basophilic cytoplasm. Mitotic figures, often irregular and atypical, were common. Immunohistochemical studies confirmed that the tumor was composed of plasma cells, as evidenced by their reactivity with antibodies to CD138, CD45, vimentin, Kappa light chain, and EMA (Fig. 4). It was negative for Lambda light chain, CD20, CD3, CD56, CD10, S-100, neuron-specific enolase, placental alkaline phosphatase, smooth muscle actin, and creatine kinase. Moreover, urine Bence-Jones protein was negative, bone marrow aspiration revealed about 4% plasma cells, and skeletal X-ray showed no lytic lesion. All of these findings contributed to a diagnosis of primary renal plasmacytoma in this case. The patient refused chemotherapy or radiotherapy and there was no recurrence or metastasis that occurred during a 22-month follow-up.

## 3 Discussion

An extramedullary plasmacytoma is a rare malignant neoplasm usually arising from the B-lymphocytes outside the bone marrow. EMP occurs either as a solitary plasmacytoma or multiple myeloma. The diagnosis of EMP depends upon the demonstration of extramedullary plasma cell tumor with no evidence of systemic signs and symptoms associated with MM. EMP commonly involves the respiratory tract, larynx, as well as the gastrointestinal tract and lymph nodes. EMP arising in the kidney is very rare and there are only few reports about EMP of kidney in the literature.^[4–9]^

Radiological findings in renal plasmacytoma are indistinguishable from renal cell carcinoma (RCC). Radiological diagnosis of EMP is infrequent owing to its location and nonspecific manifestations. The CT features of abdominal EMP show a multifocal pathology with involvement of perirenal space and are most frequently seen as well-defined enhancing masses.^[10]^ Some cases revealed soft tissue masses along the renal capsule and paraaortic region that were of relatively low attenuation accompanied by granular calcifications.^[11]^ In our case, CT attenuation of this tumor was approximately 50 HU which revealed a relatively “hyperdense” mass. This finding is seen in only 2% of RCC and hyperdense, homogeneously enhancing mass has a low possibility of being RCC. So, biopsy is recommended when looking at this kind of tumors.^[12]^ If biopsy is performed, radiotherapy may be the treatment of choice and unnecessary surgery could be avoided.

Definite diagnosis is reached only through histopathological examination coupled with immunohistochemistry. Plasmacytic tumors may be composed of pleomorphic cells with very little resemblance to normal plasma cells.^[13]^ Such tumors may be difficult to diagnose and the differential diagnosis of anaplastic tumors could be made without immunohistochemistry. Nevertheless, the differential diagnosis between EMP, lymphoma, and other kinds of tumors can sometimes be troublesome. Such atypical plasmacytomas may present diagnostic problems without immunohistochemistry. Hence, it is important to establish a methodology to differentiate anaplastic tumors, especially in unusual locations such as the kidney. The diagnostic criteria for solitary EMP is as follows: tissue with plasma cell histology; infiltration of bone marrow plasma cells is less than 5% of all nucleated cells; absence of osteolytic bone lesions or other tissue involvement.^[14]^

No guidelines are available for EMP due to its rarity and variable presentation and there are no widely established treatment criteria. Surgery or radiotherapy, alone or in combination, can be used according to tumor sizes, clinical stage, and willingness of the patient. Surgical removal of the tumor can be performed as sole treatment for small masses, particularly for a limited and easily resectable mass just like our case. EMP is highly radiosensitive and nearly all patients successfully achieve local control.^[15]^ The disease can be cured by radiotherapy, however, it is difficult to establish its role because of the small number of renal EMP patients who have received radiotherapy. Treatment with chemotherapy does not appear to be indicated because it had no effect on the course of EMP and is not recommended.^[16]^ Any risk factors for the development of MM are not described. After treatment, all patients with EMP should have close observation to detect local recurrences or progression to MM. Generally, the clinical outcome and prognosis of EMP are favorable and the overall 5-year survival rate ranges from 53% to 75%.^[1]^

## 4 Conclusion

Renal plasmacytoma has a relatively indolent clinical course and radical nephrectomy without adjuvant therapy may result in a good prognosis. Dissemination of EMP occurred in 15% of the patients and frequently within the first 3 years, thereby indicating that EMP should be followed up for a long period following surgery.
